# Petrified Forests

**Published:** 1881-10

**Authors:** 


### PETRIFIED FORESTS.

In 1871, when the writer of this spent some weeks in the Rocky Mountains, the petrified remains of a forest of redwood, oak and other trees, petrified, thrown up from a lower level by volcanic action, and deeply imbedded in tufa, still with many portions of trunks some feet above the surface, were still to be found midway between Golden

Pass and the Ute Pass, near Pike's Peak. It is now said to have disappeared, at least so far as anything is to be seen above the surface. It is said that another of these wonderful pre-historic series of remains in Sonoma, California, is fast disappearing before the zeal of relic hunters. It is to be regretted that these wonderful remains of the mysterious past could not be preserved—and it may not yet be too late for the State governments in which they are to be found to do something towards that end. The one in California must have been buried very deep by the volcanic dust, as at the time the writer refers to one of the trunks was hollow, and a string and a stone at the end was let down and found to go many feet below the surface. No doubt if this old forest could be dug out to the original surface of the ground, many interesting relics of plants and animals might be brought to light. Some exposed strata near, thrown up at the time the trees were destroyed, exhibited numerous skeletons of fish, showing that life at least of a high order of creation existed when these redwood trees were growing there. There are now no redwood trees living in Colorado, nor any oaks beyond one shrubby species, Quercus undulata.—*Gardener's Monthly.*

